# Glycolic Acid-Catalyzed Deamidation of Asparagine Residues in Degrading PLGA Matrices: A Computational Study

**DOI:** 10.3390/ijms16047261

**Published:** 2015-03-31

**Authors:** Noriyoshi Manabe, Ryota Kirikoshi, Ohgi Takahashi

**Affiliations:** Faculty of Pharmaceutical Sciences, Tohoku Pharmaceutical University, 4-4-1 Komatsushima, Aoba-ku, Sendai 981-8558, Japan; E-Mails: manabe@tohoku-pharm.ac.jp (N.M.); kirikoshi@tohoku-pharm.ac.jp (R.K.)

**Keywords:** peptide and protein drugs, asparagine residue, deamidation, succinimide, PLGA, glycolic acid catalysis, computational chemistry, double proton transfer, concerted bond reorganization

## Abstract

Poly(lactic-*co*-glycolic acid) (PLGA) is a strong candidate for being a drug carrier in drug delivery systems because of its biocompatibility and biodegradability. However, in degrading PLGA matrices, the encapsulated peptide and protein drugs can undergo various degradation reactions, including deamidation at asparagine (Asn) residues to give a succinimide species, which may affect their potency and/or safety. Here, we show computationally that glycolic acid (GA) in its undissociated form, which can exist in high concentration in degrading PLGA matrices, can catalyze the succinimide formation from Asn residues by acting as a proton-transfer mediator. A two-step mechanism was studied by quantum-chemical calculations using Ace-Asn-Nme (Ace = acetyl, Nme = NHCH_3_) as a model compound. The first step is cyclization (intramolecular addition) to form a tetrahedral intermediate, and the second step is elimination of ammonia from the intermediate. Both steps involve an extensive bond reorganization mediated by a GA molecule, and the first step was predicted to be rate-determining. The present findings are expected to be useful in the design of more effective and safe PLGA devices.

## 1. Introduction

Poly(lactic-*co*-glycolic acid) (PLGA) is a polyester copolymer of lactic acid (LA) and glycolic acid (GA), which has been approved for drug delivery use by the United States Food and Drug Administration (FDA) owing to its biocompatibility and biodegradability [1,2,3]. PLGA has been extensively studied, in particular for the controlled release of peptide and protein drugs. However, chemical degradation reactions of peptides and proteins encapsulated in PLGA have been reported [4,5,6,7,8,9], which are important concerns in the design and development of PLGA-based formulations of peptides and proteins.

PLGA is initially a hydrophobic polymer, necessitating the use of organic solvents for formulation. In aqueous solution, or under moisture stress, it undergoes hydrolysis to produce monomers and oligomers of LA and GA. Their accumulation inside PLGA matrices (microspheres or films) produces an acidic microclimate therein [5,10,11,12], which can induce various degradation reactions of the encapsulated peptides and proteins [5,6,7,8,9]. The microclimate pH within degrading PLGA matrices can indeed be as low as 1.5 [11]. Deamidation reactions of asparagine (Asn or N) residues are among those reactions that have been reported to occur in degrading PLGA matrices [4,5,7,8,9].

**Scheme 1:**
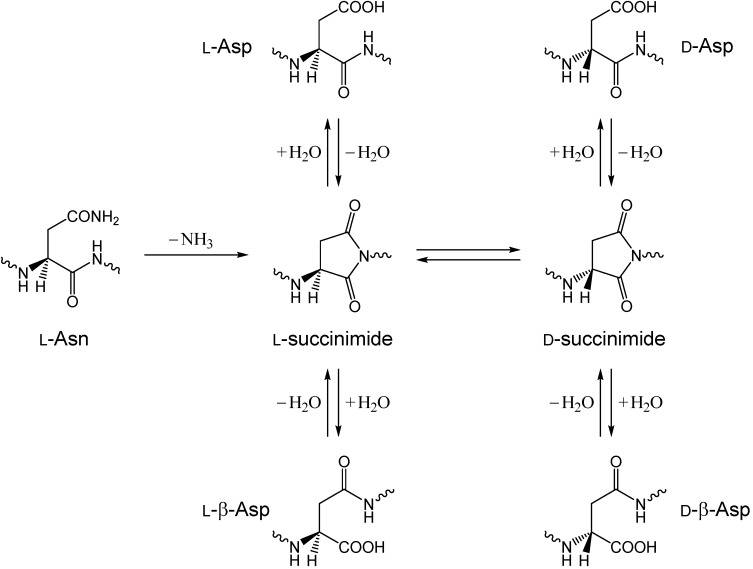
Succinimide-mediated deamidation of asparagine (Asn) residues producing aspartic acid (Asp) and β-Asp residues.

Asn deamidation reactions, occurring nonenzymatically or spontaneously in peptides and proteins, have been extensively studied in many fields of chemical and pharmaceutical sciences [13,14,15,16,17,18]. In acidic aqueous solution (pH 1–2, HCl buffer), Asn deamidation in model peptides has been shown to occur by acid-catalyzed direct hydrolysis of the side-chain amide group, converting the Asn residue to an aspartic acid (Asp) residue [19,20]. At pHs higher than 5, however, a succinimide-mediated mechanism as shown in Scheme 1 has been well established [14,17,18,21,22]. In this mechanism, a succinimide, which results from intramolecular nucleophilic attack of the backbone amide nitrogen of the following residue on the side-chain amide carbon, is the immediate product, which then undergoes hydrolysis either to an Asp or a β-Asp residue, typically in a ratio of approximately 1:3 for peptides. Moreover, since the succinimide intermediate is racemization-prone, d-Asp or d-β-Asp residue may also be produced via d-succinimide [20,21,23]. It should also be noted that pharmaceutical potentials of α-aminosuccinimides have just started to be fully recognized [24].

Here, it is very interesting to note that, in degrading PLGA films, an Asn-containing peptide VYPNGA (V = valine, Y = tyrosine, P = proline, G = glycine, A = Alanine) has been shown to undergo deamidation to a succinimide along with that to the corresponding Asp-containing peptide [8]. It is noteworthy that no β-Asp-containing peptide was observed as a deamidation product. Considering that succinimides are acid-stable [19,25], it is implied that, in degrading PLGA, direct formation of succinimide from Asn occurs by a novel mechanism in addition to direct hydrolysis of the Asn side-chain amide group. In this paper, we computationally show that GA in its undissociated form, CH_2_(OH)COOH, can catalyze succinimide formation from Asn residues. Note that the p*K*_a_ of GA is 3.83, meaning that GA is mainly present in the undissociated form at pH lower than 3. Therefore, the concentration of the undissociated form of GA can be very high inside degrading PLGA matrices.

The idea of catalysis by GA is based on our recent computational study of an acetic acid (AA)-catalyzed mechanism of succinimide formation from Asp residues [26], where an AA molecule acts as a proton-transfer mediator in cyclic hydrogen-bonded complexes, leading to an extensive bond reorganization. By this mechanism, it was successfully explained why the backbone amide nitrogen can make a bond to the side-chain carboxyl carbon despite of its poor nucleophilicity. It should also be noted that, very recently, various carboxylic acids including GA have been shown to catalyze deamidation of a hexapeptide YGKNGG (K = lysine) in solution, although the catalytic mechanism was not revealed [27].

**Scheme 2:**
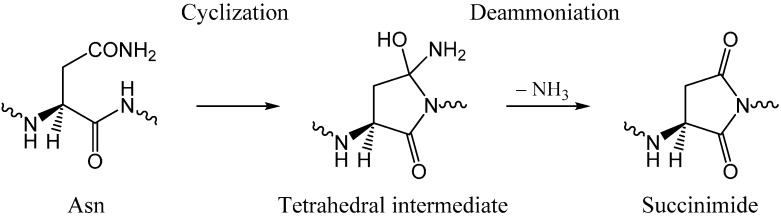
Two-step mechanism for succinimide formation from an Asn residue.

**Figure 1:**
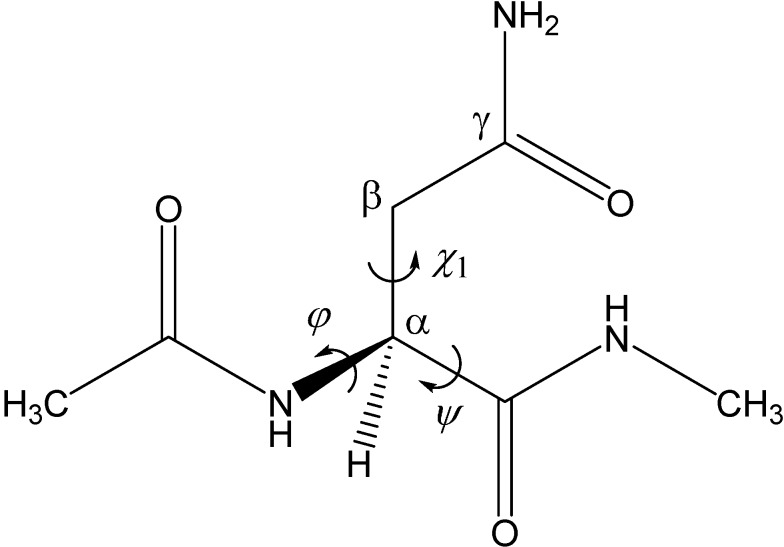
The model compound used in the present study (Ace-Asn-Nme, where Ace = acetyl and Nme = NHCH_3_). The φ (C–N–C_α_–C) and ψ (N–C_α_–C–N) dihedral angles, which characterize the main-chain conformation, and the χ_1_ dihedral angle (N–C_α_–C_β_–C_γ_), which characterizes the side-chain conformation, are indicated.

As shown in Scheme 2, succinimide formation from an Asn residue is comprised of two steps (an addition-elimination or a cyclization-deammoniation mechanism). In the first step, a so-called tetrahedral intermediate is formed by nucleophilic attack of the amide nitrogen of the *C*-terminal peptide bond on the amide carbon of the Asn side chain. In the second step, an ammonia molecule is eliminated from the intermediate to give a succinimide molecule. The mechanism by which a GA molecule catalyzes this two-step reaction has been investigated using Ace-Asn-Nme (Ace = acetyl, Nme = NHCH_3_) (Figure 1) as a model compound as in related previous studies [28,29].

## 2. Results and Discussion

**Figure 2:**
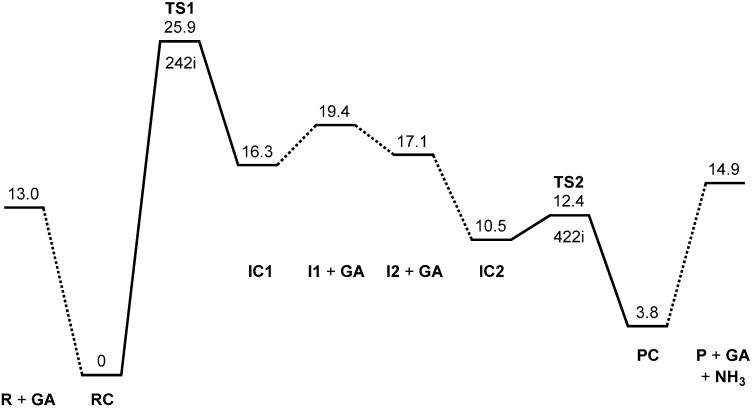
Energy diagram, where MP2 (second-order Møller-Plesset perturbation theory) relative energies corrected for the zero-point energy (ZPE) and the SM8 (solvation model 8) hydration free energy are shown in kcal·mol^−1^ (see text for more details). R, reactant; GA, glycolic acid; RC, reactant complex; TS, transition state; I, intermediate; IC, intermediate complex; P, product; PC, product complex. The imaginary frequency (cm^−1^) is also shown for TS1 and TS2.

**Figure 3:**
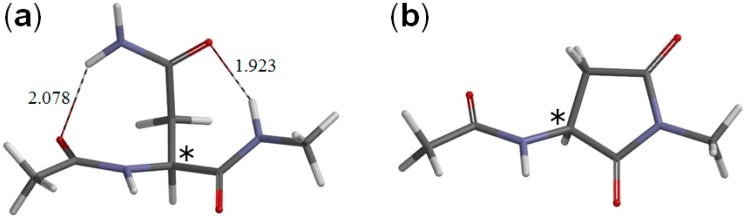
The geometries of (**a**) the reactant R (model compound, Figure 1) (φ = 172°, ψ = −133°, χ_1_ = 59°) and (**b**) the succinimide product P (φ = −172°, ψ = −141°, χ_1_ = 136°). The asterisk (*) indicates the α carbon. Hydrogen bond distances are shown in Å.

**Figure 4:**
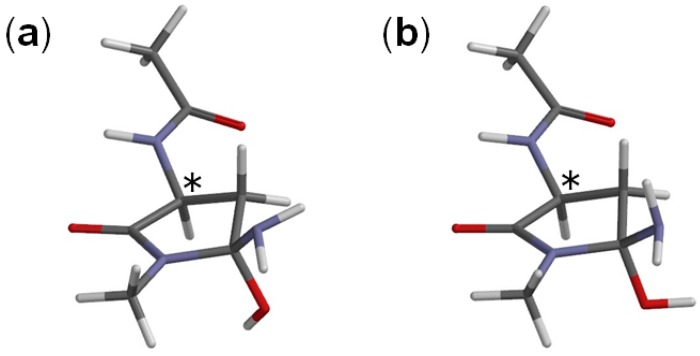
The geometries of two conformers of the tetrahedral intermediate; (**a**) I1 (φ = −171°, ψ = −145°, χ_1_ = 146°) and (**b**) I2 (φ = −174°, ψ = −144°, χ_1_ = 148°). The asterisk (*) indicates the α carbon.

**Figure 5:**
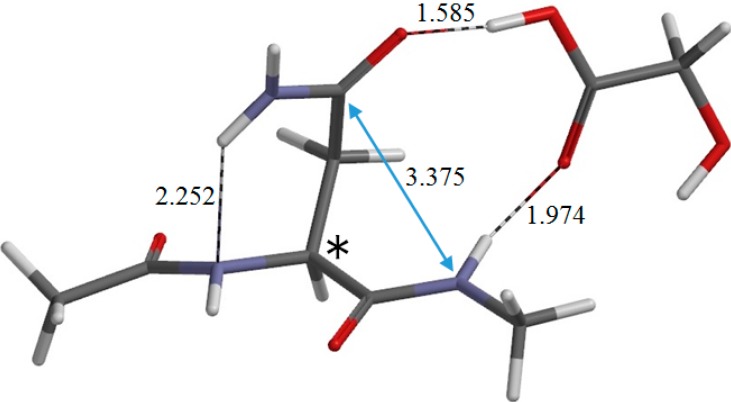
The geometry of the reactant complex RC (φ = −158°, ψ = −170°, χ_1_ = 71°). The asterisk (*) indicates the α carbon. Selected interatomic distances are shown in Å.

**Figure 6:**
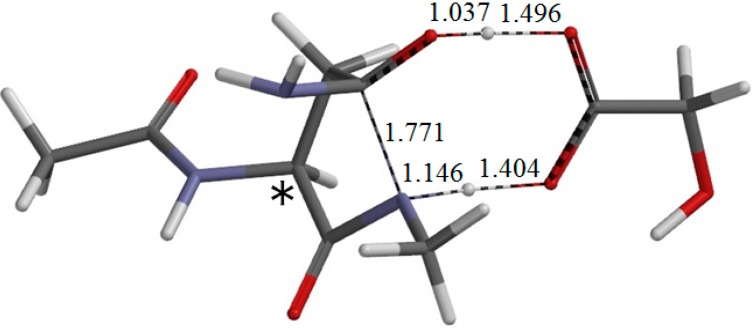
The geometry of the first-step transition state TS1 (φ = −168°, ψ = −143°, χ_1_ = 105°). The asterisk (*) indicates the α carbon. The distances of forming and breaking bonds are shown in Å.

**Figure 7:**
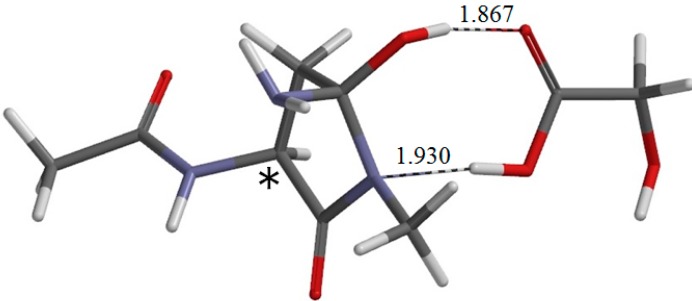
The geometry of IC1 (φ = −167°, ψ = −133°, χ_1_ = 108°), which is the intermediate complex directly connected to TS1. The asterisk (*) indicates the α carbon. Hydrogen bond distances are shown in Å.

**Figure 8:**
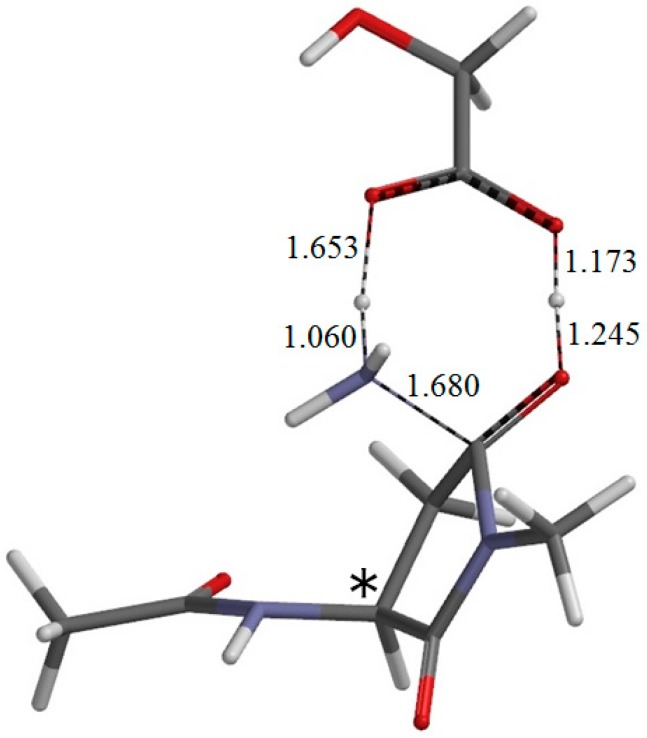
The geometry of IC2 (φ = −174°, ψ = −116°, χ_1_ = 101°), which is the intermediate complex directly connected to TS2 (the second-step transition state shown in Figure 9). The asterisk (*) indicates the α carbon. Hydrogen bond distances are shown in Å.

**Figure 9:**
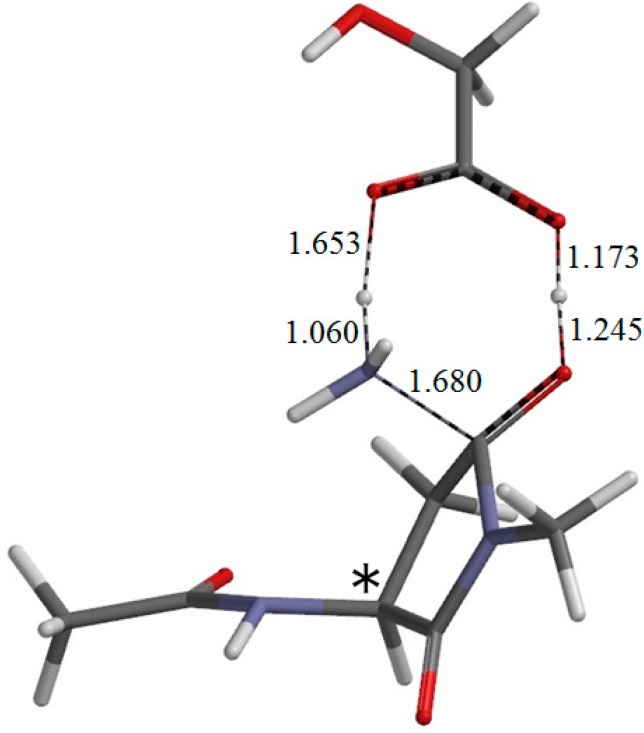
The geometry of the second-step transition state TS2 (φ = −172°, ψ = −112°, χ_1_ = 96°). The asterisk (*) indicates the α carbon. The distances of forming and breaking bonds are shown in Å.

**Figure 10:**
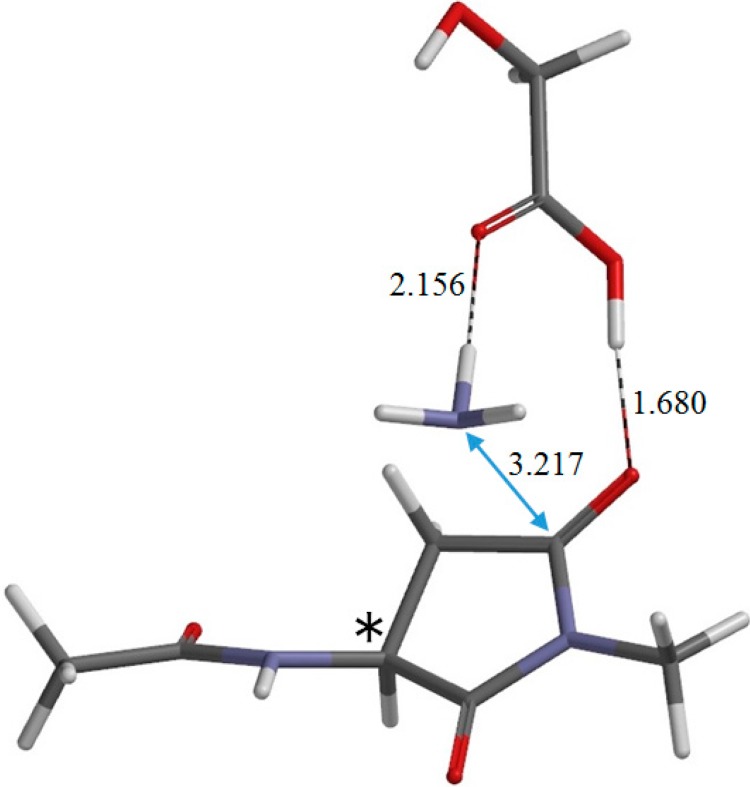
The geometry of the product complex PC (φ = −165°, ψ = −144°, χ_1_ = 137°). The asterisk (*) indicates the α carbon. Selected interatomic distances are shown in Å.

Figure 2 shows the energy diagram for the two-step succinimide formation catalyzed by a GA molecule, CH_2_(OH)COOH, and Figure 3, Figure 4, Figure 5, Figure 6, Figure 7, Figure 8, Figure 9 and Figure 10 show optimized geometries. The values of dihedral angles φ, ψ and χ_1_ (Figure 1) are shown in the captions to Figure 3, Figure 4, Figure 5, Figure 6, Figure 7, Figure 8, Figure 9 and Figure 10. While geometry optimizations and zero-point energy (ZPE) calculations were performed by the B3LYP density functional theory (DFT) method, single-point energy calculations at the optimized geometries were performed at the MP2 (second-order Møller-Plesset perturbation theory) level of theory to obtain more reliable energetics. Moreover, hydration free energies were estimated by single-point B3LYP calculations using the SM8 (solvation model 8) continuum model [30,31]. The 6-31+G(d,p) basis set was used throughout. All relative energies reported in this work are calculated using the MP2 energies and corrected for the ZPEs and SM8 hydration free energies.

The optimized reactant molecule R (model compound) shown in Figure 3a has a conformation in which the backbone is extended (φ = 172°, ψ = −133°) and has two intramolecular hydrogen bonds between the Asn side chain and the backbone. Figure 5 shows the reactant complex RC (φ = −158°, ψ = −170°) formed between R and a glycolic acid molecule GA. Upon the formation of RC, the hydrogen bond (1.923 Å) between the NH hydrogen of the Nme group and the oxygen of the Asn side chain in R is broken. Instead, two new hydrogen bonds are formed between R and GA; one is between the NH hydrogen and the C=O oxygen of GA (1.974 Å), and the other is between the carboxyl hydrogen of GA and the C=O oxygen of the Asn side chain (1.585 Å). Moreover, the hydrogen bond (2.078 Å) between the oxygen of the Ace group and one of the NH_2_ hydrogens in R is broken, while the latter forms a new weak hydrogen bond (2.252 Å) to the backbone nitrogen of the Asn residue. The complexation energy between R and GA is 13.0 kcal·mol^−1^.

From RC, cyclization occurs via the transition state TS1 (the transition state of the first step) (Figure 6) to give an intermediate complex IC1 (the intermediate complex directly connected to TS1) (Figure 7). In this step, a new single bond is formed between the nitrogen of the Nme group and the amide carbon of the Asn side chain to form a five-membered ring. The distances between these two atoms are 3.375, 1.771, and 1.504 Å in RC, TS1, and IC1, respectively.

The energy of TS1 relative to the reactant complex RC is 25.9 kcal·mol^−1^. Because the energy of TS2 (the transition state of the second step) is much lower (see below), the first step is predicted to be rate determining. Very recently, activation energies for the deamidation of a hexapeptide YGKNGG in the presence of various carboxylic acids including GA have been reported to be 21.4–24.2 kcal·mol^−1^ [27]. The presently calculated activation barrier of 25.9 kcal·mol^−1^ is consistent with these experimental values, which implies that the mechanism proposed here actually operates in degrading PLGA matrices. Moreover, a similar mechanism may also operate in the above-mentioned deamidation reactions of YGKNGG [27].

Concomitantly with the C–N bond formation, a double proton transfer mediated by the GA molecule occurs, so that the resultant tetrahedral intermediate has NH_2_ and OH groups on the C_γ_ atom. More specifically, the NH hydrogen moves toward the C=O oxygen of GA, the carboxyl hydrogen of GA moves toward the C=O oxygen of the side chain, and the single and double bonds are interchanged in the COO moiety of GA. The GA molecule thus acts as both proton donor and acceptor in the double proton transfer. In the resultant intermediate complex IC1, the newly-formed GA molecule forms two hydrogen bonds to the intermediate molecule. One is between the amide nitrogen in the five-membered ring and the carboxyl OH of GA (1.930 Å); the other is between the C=O of GA and the newly-formed OH on the five-membered ring (1.867 Å). When IC1 is formed from RC via TS1, the dihedral angles ψ and χ_1_ change by about 37° because of the ring formation, while change in φ is much smaller.

When the GA molecule was removed from IC1 and the remaining intermediate moiety was geometry-optimized, I1 (intermediate conformer related to IC1) shown in Figure 4a was obtained. The complexation energy between I1 and GA is 3.1 kcal·mol^−1^. For the second step to occur, the catalytic GA molecule has to now form hydrogen bonds to both of the NH_2_ and OH groups on the C_γ_ atom as in IC2 (Figure 8), which is the intermediate complex directly connected to the second-step transition state (TS2) shown in Figure 9. In IC2, the distance of the hydrogen bond between the NH_2_ nitrogen and the carboxyl hydrogen of GA is 1.671 Å, and that between the OH hydrogen of the intermediate molecule and the C=O oxygen of GA is 1.777 Å. When the GA molecule was removed from IC2 and the remaining intermediate moiety was geometry-optimized, I2 (intermediate conformer related to IC2) shown in Figure 4b was obtained. The complexation energy between I2 and GA is 6.6 kcal·mol^−1^. IC2 is lower in energy than IC1 by 5.8 kcal·mol^−1^, which can be attributed to stronger hydrogen bonds than in IC1, as may be seen from Figure 7 and Figure 8.

From IC2, elimination of ammonia occurs via TS2 to give the product complex PC (Figure 10). The energy of TS2 relative to RC is 12.4 kcal·mol^−1^, which is lower than that of TS1 by 13.5 kcal·mol^−1^. The local activation barrier of the second step is only 1.9 kcal·mol^−1^. In this step, the C–N bond on the five-membered ring is cleaved. In TS2, the distance of the breaking C−N bond is elongated to 1.680 from 1.490 Å in IC2. Concomitantly with this bond cleavage, a double proton transfer mediated by GA occurs. The carboxyl hydrogen of GA moves toward the departing nitrogen, leading to formation of an NH_3_ molecule. On the other hand, the hydrogen atom attached to the oxygen atom on the five-membered ring moves toward the C=O oxygen of GA. In this double proton transfer, the GA molecule again acts as both proton donor and acceptor. It is seen from Figure 9 that the protonation of the NH_2_ group has almost completed at TS2. It can be said that the proton transfer to the NH_2_ group precedes the C–N bond cleavage and the other proton transfer.

The resultant PC is a complex formed between the succinimide product P (Figure 3b), GA, and an ammonia molecule NH_3_, and its backbone is in an extended conformation (φ = −165°, ψ = −144°). When PC is formed from IC2 via TS2, changes in ψ and χ_1_ are much larger than in φ. In PC, the C=O oxygen and carboxyl hydrogen of GA form hydrogen bonds to NH_3_ (2.156 Å) and one of the carbonyl groups of the succinimide moiety (1.680 Å), respectively. The complexation energy for the formation of PC from separated P, GA, and NH_3_ is 11.1 kcal·mol^−1^. When the energies of the initial separated state (R + GA) and the final separated state (P + GA + NH_3_) are compared, the latter is higher by 1.9 kcal·mol^−1^.

From a mechanistic point of view, both the first and second steps involve an extensive, concerted bond reorganization occurring in a cyclic hydrogen-bonded complex, as in the AA-catalyzed succinimide formation from Asp residues which we have recently proposed computationally [26].

## 3. Computational Details

Figure 1 shows the model compound used in the present study, in which an Asn residue is capped with Ace and Nme groups on the *N*- and *C*-termini, respectively. This compound was previously used in related computational studies by Catak *et al.* [28,29]. In addition, its Asp counterpart has recently been used in our computational study of an AA-catalyzed mechanism of succinimide formation from Asp residues [26]. The two-step reaction pathway (cyclization-deammoniation, Scheme 2) was explored for a reactant complex formed between the model compound and a catalytic GA molecule.

All calculations were performed by using Spartan’14 [32]. As in our previous study [26], energy-minimum and transition state geometries were located in a vacuum without any constraints by the DFT method with the B3LYP functional and the 6-31+G(d,p) basis set. Vibrational frequency calculations were performed for all of the optimized geometries to confirm them as energy minima (with no imaginary frequency) or transition states (with a single imaginary frequency), and to obtain ZPEs. Intrinsic reaction coordinate (IRC) calculations were performed from the transition states followed by full geometry optimizations to confirm that each transition state connects two energy minima, as shown in Figure 2 by solid lines. Moreover, single-point MP2 calculations have been performed using the 6-31+G(d,p) basis set at all the optimized geometries to obtain more reliable energetics. Finally, hydration free energies have been estimated by employing the SM8 continuum model [30,31] at the B3LYP/6-31+G(d,p) level. All relative energies reported in this work have been calculated using the MP2 total energies and corrected for the ZPEs and SM8 hydration free energies calculated at the B3LYP/6-31+G(d,p) level of theory.

## 4. Conclusions

It has been computationally shown that glycolic acid in its protonated form (GA) can catalyze succinimide formation from Asn residues. The reaction is comprised of two steps, cyclization to form the tetrahedral intermediate and its deammoniation. A GA molecule can catalyze both steps by acting as both proton donor and acceptor in double proton transfers, and the rate-determining step was predicted to be the first step. The GA-catalyzed mechanism of Asn deamidation can operate in degrading PLGA matrices, where GA molecules exist in high concentration. Lactic acid (LA, p*K*_a_ = 3.86) is also expected to catalyze the Asn deamidation by a similar mechanism. These findings are expected to be useful in the design of more effective and safe PLGA-based controlled-release devices. From the viewpoint of mechanistic organic chemistry, reaction mechanisms involving carboxylic acid-mediated bond reorganization are expected to be more general.
